# Varicella-Zoster Virus Reactivation and Increased Vascular Risk in People Living with HIV: Data from a Retrospective Cohort Study

**DOI:** 10.3390/v15112217

**Published:** 2023-11-06

**Authors:** Deborah Fiordelisi, Mariacristina Poliseno, Nicolo’ De Gennaro, Eugenio Milano, Carmen Rita Santoro, Francesco Vladimiro Segala, Carlo Felice Franco, Giorgia Manco Cesari, Luisa Frallonardo, Giacomo Guido, Giuliana Metrangolo, Greta Romita, Francesco Di Gennaro, Annalisa Saracino

**Affiliations:** 1 Clinic of Infectious Diseases, Department of Precision and Regenerative Medicine and Ionian Area, Polyclinic of Bari, University Hospital Polyclinic, University of Bari, Piazza Giulio Cesare n. 11, 70124 Bari, Italy; deborah.fiordelisi88@gmail.com (D.F.); carlofelicefranco@libero.it (C.F.F.);; 2 Clinic of Infectious Diseases, Department of Medical and Surgical Sciences, Policlinic of Foggia, University of Foggia, Viale Luigi Pinto n. 1, 71122 Foggia, Italy

**Keywords:** Varicella–zoster virus, VZV, HIV, stroke, vascular risk

## Abstract

Background: The increased vascular risk associated with varicella–zoster virus (VZV) reactivation is extensively established in the general population. This retrospective cohort study investigates whether this observation holds for People Living with HIV (PLWH), a group already confronting heightened cardiovascular risk. Methods: Among PLWH who initiated antiretroviral therapy (ART) at our center and have been under our care for >24 months since 1st January 2005, individuals with a history of herpes zoster (HZ) were identified, and their features were compared with those of PLWH with no history of HZ. The prevalence of ischemic events (deep venous thrombosis, stroke, and acute myocardial infarction) was calculated and compared using the chi-square test. An odds ratio (O.R.) and a 95% confidence interval (C.I.) for ischemic events following HZ were evaluated through univariate and multivariate logistic regression. Results: Overall, 45/581 PLWH reported HZ. Ischemic events followed HZ significantly more often than not (13% vs. 5%, *p* = 0.01). Positive serology for both VZV and HZ correlated with increased ischemic risk (O.R. 4.01, 95% C.I. 1.38–11.6, *p* = 0.01 and O.R. 3.14, 95% C.I. 1.12–7.68, *p* = 0.02, respectively), though chronic heart disease demonstrated stronger predictive value in multivariate analysis(O.R. 8.68, 95% C.I. 2.49–29.50, *p* = 0.001). Conclusions: VZV potentially exacerbates vascular risk in PLWH, particularly in the presence of other predisposing factors. Further research is needed to confirm our data.

## 1. Introduction

Herpes zoster (HZ) is defined as a painful, erythematous, maculopapular rash associated with fluid-filled lesions resulting from reactivation of the varicella–zoster virus (VZV), which lies dormant in the spinal and cranial sensory ganglia after primary infection in childhood. The unilateral presentation and restriction to a single dermatome are unique features that distinguish HZ from other dermatological rashes [1]. The calculated cumulative occurrence of this phenomenon is approximately six instances for every 1000 individuals in North America and Europe. Despite the availability of a recombinant zoster vaccine (Shingrix, GlaxoSmithKline, Research Triangle Park, NC, USA), which is recommended at least for adults aged 50 years and older, data from the past decade reveal that this occurrence rate is consistently increasing [2,3]. In addition to older age and female sex, family history, physical trauma, and comorbidities such as diabetes, rheumatoid arthritis, cardiovascular disease, renal disease, systemic lupus erythematosus, and inflammatory bowel disease have been included among the main risk factors for HZ. Immunosuppression, in particular, due to malignancies or human immunodeficiency virus/acquired immune deficiency syndrome (HIV/AIDS) has been associated with a significantly increased risk of VZV reactivation [4].

Serious and common complications of HZ include acute, chronic, and/or recurrent keratitis and iritis, neurotrophic keratopathy, and postherpetic neuralgia [3].

A less common aspect of the phenomenon concerns the association between VZV reactivation and increased vascular risk. Recent studies conducted in the general population have indeed brought attention to a connection between HZ and the occurrence of short- and medium-term ischemic cardiac and cerebral events, particularly during the initial three months after reactivation. This association is particularly significant in individuals under the age of 50 and in those who develop ophthalmic herpes zoster [5,6,7]. There is only limited evidence available regarding the occurrence of this phenomenon in people living with HIV (PLWH), even though VZV reactivation is quite common among this group, particularly when there is a significant depletion of CD4+ cells [8,9]. Furthermore, compared to the general population, PLWH frequently face an increased risk of cardiovascular issues, largely due to various contributing factors [10]. In light of this, a retrospective analysis was conducted using our databases to explore the potential link between HZ and increased vascular risk in a single-center cohort of PLWH who are receiving antiretroviral treatment (ART). The goal was to ascertain whether there exists an association between both documented VZV infection and HZ clinical manifestation and the occurrence of any ischemic events within this specific population. Additionally, the study aimed to determine the extent to which chronic VZV infection contributes to heightened vascular risk in this special group, in comparison to other factors regarding patients’ history of HIV infection, relative medication use, and other coexisting, non-communicable health conditions.

## 2. Materials and Methods

### 2.1. Inclusion Criteria and Data Collection

This was a retrospective observational cohort study conducted at a single center. It included all individuals with HIV-1 infection who, as of 30 June 2023, met the following criteria, starting from 1 January 2005:

(i) Age ≥ 18 years;
(ii) Initiated ART at the Infectious Diseases Clinic of the Policlinic of Bari;
(iii) Were under care at our center for a minimum of 24 months;
(iv) Provided written informed consent for the collection and use of clinical data for research purposes at the time of initiating ART.

For these patients, sociodemographic characteristics (sex, age, nationality, and mode of HIV transmission), immunovirological and therapeutic information related to HIV infection (duration of HIV infection, AIDS diagnosis at baseline, nadir of CD4 cell count, duration of ART, history of abacavir use, and current antiretroviral regimen), and current comorbidities (hypertension, type II diabetes, dyslipidemia, and chronic heart disease) were retrospectively collected from the computer system used at the clinic. This system is continuously updated as patients receive follow-up care, including the recording of laboratory test results. It also includes a dedicated section for documenting medical visits, and this section is meticulously updated by the medical staff. Our research involved a retrospective analysis of both the results of laboratory tests and the records of medical visits. Cases of HZ were identified based on their clinical descriptions. For instances of HZ diagnosed clinically, we documented specific details, such as the location of the reactivation on the body, the frequency of recurring episodes, and the patient’s history of vaccination.

Data regarding VZV infections or reactivations were collected for analysis. The presence of positive immunoglobulin G(IgG) serology for VZV was utilized as an indicator of prior infection. Detailed information regarding instances of VZV reactivation was gathered, and in cases of HZ, details such as the location of bodily reactivation, frequency of recurring episodes, and vaccination history were documented.

Subsequently, the medical records of the patients were scrutinized to identify any occurrences of ischemic events. Ischemic events included cerebral ischemia or stroke, acute myocardial infarction (AMI), and peripheral thrombosis.

Regarding stroke patients, only those who had events in which a cardiac origin had been ruled out were considered. In cases where patients experienced a combination of two or more of the aforementioned ischemic events, we considered the event that occurred first in the analysis to prevent fragmentation. Moreover, in PLWH with a history of VZV reactivation, only ischemic events that were documented after the date of the initial manifestation of HZ were taken into account. In such cases, the specific type of event, its outcome, and the time interval from the onset of HZ manifestation were recalled and considered for analysis.

### 2.2. Statistical Analysis

The prevalence, expressed according to the patient population, was evaluated for:

(i) Patients with positive VZV serology.
(ii) Patients with a clinical history of at least one episode of VZV reactivation.

The clinical characteristics of this group are described in terms of absolute numbers and percentages (%) for categorical variables and the median and interquartile range (IQR) for continuous variables. These variables were compared with the corresponding variables of the group of patients with a negative history of VZV reactivation. For categorical variables, the chi-square test or Fisher’s exact test was used. The distribution of continuous variables was assessed using normality tests such as Shapiro-Wilk’s test. The non-parametric Mann-Whitney U-test was utilized to test the null hypothesis of no difference between continuous non-parametric variables between the two study groups.

The prevalence, expressed as a ratio, of ischemic events (cerebral ischemia or stroke, AMI, and peripheral thrombosis)was evaluated and compared between the VZV group and the control group via the chi-square test.

This study aimed to determine the increased likelihood of experiencing a thrombotic or ischemic event in individuals with positive serology for VZV. This assessment involved quantifying the association using the odds ratio (O.R.), accompanied by a 95% confidence interval (C.I.) to indicate the precision of the estimates. The same analysis was applied while considering the medical history of clinical HZ manifestations. Furthermore, the analysis was conducted separately to assess the risk of the following specific outcomes: (i) the occurrence of any type of ischemic/thrombotic event and (ii) the incidence of stroke, AMI, or peripheral thrombosis about other forms of vascular events. In this scenario, patients who experienced two or more of the aforementioned ischemic or thrombotic events were treated as separate entries in each respective group.

Finally, a multivariate model was developed to assess the factors that could predispose a patient to a higher vascular risk, in addition to a positive history of VZV reactivation. The variables considered in this model included male sex, age over 65 years, history of endovascular drug use, nadir CD4 count below 200, history of antiretroviral and HIV therapy exceeding 10 years, positive pharmacological history for abacavir, current use of protease inhibitor (PI)-based regimens, hypertension, type II diabetes, dyslipidemia, and chronic heart disease. These variables were selected based on a combination of evidence from the existing literature and the results of the chi-square test, focusing on the variables that showed significant differences in the descriptive analysis comparing the two study groups. The multivariate model incorporated all variables that demonstrated a *p*-value of less than 0.2 following assessment in the univariate analysis.

The analyses were performed using Jamovi version 2.3.

The level of statistical significance was set at *p* < 0.05 for all analyses.

## 3. Results

As of 30 June 2023, a total of 581 patients were included, mainly males (81%), with a median age of 48 (38–56) years.

All patients were tested for VZV serology at least once. Positive serology was reported by 371 patients (74%).

Of these patients, 45 (7% of the total) also referred to at least one physical manifestation of VZV reactivation in their clinical history. The reactivation of VZV was observed most frequently in the thoracodorsal region (17 patients, 39%), followed by the lumbosacral (7 patients, 15%), facial (5 patients, 11%), and upper (4 patients, 9%) and lower limb (3 patients, 7%) regions. In two patients (4%), a multidermatomal presentation was observed.

Eleven patients (2% of the total) had received vaccination against VZV, two due to a history of recurrent HZ manifestations. For the other patients, data were missing.

Compared to PLWH without VZV reactivation, PLWH who experienced VZV reactivation were notably older, with a lengthier history of HIV infection and ARV treatment. Additionally, among this group, a greater prevalence of individuals with a baseline AIDS diagnosis and various comorbidities (particularly hypertension and dyslipidemia) was observed (Table 1).

The overall incidence rate of ischemic events was 5% (31 subjects). Notably, the occurrence of an ischemic event was significantly more frequent among patients who also reported VZV reactivation (13% vs. 5% in the group without VZV reactivation, *p* = 0.01).

Among the 13 cases of peripheral thrombosis, (i) in five cases, deep venous thrombosis (DVT) affected the veins of the lower extremities; (ii) in one case, it was intestinal ischemia related to arterial thrombosis; (iii) in one case, it was clitoral vein thrombosis; (iv) in one case, it was central retinal vein thrombosis; and (v) in four cases, it was pulmonary artery thrombosis.

While thrombosis prevailed in the general population, stroke occurred more frequently among PLWH who experienced HZ, although this correlation was not statistically significant (50% compared to 20% in the group without VZV reactivation, *p* = 0.21).

Characteristics of six patients with a history of VZV reactivation who reported at least one ischemic event are described in Table 2.

Two patients reported an ischemic event 7 and 13 years after the reactivation of VZV, respectively. In two other patients, ischemia occurred following herpes zoster within 45 days and 5 months, respectively. Lastly, for two subjects, although it was known that the ischemic event followed VZV reactivation, precise information about the event date was unavailable, thus making it impossible to calculate the temporal interval.

Among patients who reported any ischemic event, 13 out of 31 (42%) were on ART at the time of the ischemic event. It is noteworthy that all individuals who experienced an ischemic event survived. As of the time of writing, 10 patients had passed away, but their deaths were attributed to causes unrelated to ischemic events.

In the univariate logistic regression analysis, an increased risk of developing any ischemic or thrombotic event was observed in patients with positive serology for VZV infection (O.R. 4.01, 95% C.I. 1.38–11.6, *p* = 0.01).

The same was noticed in patients with a history of HZ manifestations (O.R. 3.14, 95% C.I. 1.12–7.68, *p* = 0.02). However, no significant correlations were found between a history of VZV reactivation and increased risk of stroke (O.R. 5.71, 95% C.I. 0.82–38.3, *p* = 0.06), AMI (O.R. 2.38, 95% C.I. 0.40–14.4, *p* = 0.34), or DVT (O.R. 0.54, 95% C.I. 0.08–3.45, *p* = 0.51) when considered separately, probably due to the paucity of our population sample.

In the multivariate analysis, which was adjusted for factors including age, sex, duration of HIV infection, duration of ART, and major comorbidities, the presence of chronic heart disease emerged as the sole factor significantly elevating vascular risk in PLWH (O.R. 9.95, 95% C.I. 2.15–45.46, *p* = 0.003). Nevertheless, taking out statistical significance, that could not have been reached due to the paucity of the sample size; thus, it is interesting that a positive association with hypertension, older age, and a history of VZV infection and reactivation could still be observed after adjusting for confounding factors (Table 3).

## 4. Discussion

In people living with HIV, herpes zoster can be categorized as an opportunistic infection. In this population, its occurrence is not only more prevalent but also more likely to be accompanied by complications compared to the general population, even among individuals who are receiving effective ART [11,12]. Although limited data are available on the current seroprevalence of VZV infection in PLWH, some studies indicate a prevalence of VZV-positive serological patients of approximately 95% [13].

In our study, we observed that the prevalence of positive serology for VZV exceeded 70%. Among this group of patients, approximately 12% presented with clinical manifestations of VZV reactivation. Subjects who experienced VZV reactivation were more likely to be older, have a higher number of comorbidities, and have a longer and more complex clinical history of HIV infection.

In addition to causing various complications, such as post-herpetic neuralgia, myelitis, and meningoencephalitis, which can significantly reduce the quality of life, especially when recurrent outbreaks occur [3,14], several studies have provided evidence that VZV infection can elevate the risk of cardiovascular and cerebral ischemic events [15,16,17].

These phenomena can manifest years after an episode of HZ and are linked to VZV vasculopathy, resulting from the persistent vascular changes after a VZV infection and caused by the virus. This condition is characterized by (i) the production of prothrombotic autoimmune antibodies; (ii) autoimmune reactions triggered by immune complexes circulating in the bloodstream; and (iii) structural changes within blood vessels, including disruption of the internal elastic lamina and intimal hyperplasia and a decrease in smooth muscle cells in the tunica media layer [7,18].

In a recent study by Ku et al. [19], a 1.85-fold increased risk of stroke was observed in patients with HZ among those with HIV infection. A significantly elevated risk of stroke was found in PLWH who also had hypertension, heart disease, chronic kidney disease, hyperlipidemia, and HCV and were on treatment with protease inhibitors.

In our retrospective analysis, we found that the overall prevalence of ischemic events was approximately 5% among a cohort of over 500 PLWH who had been followed up since the initiation of ART for a median duration of eight years. Our data revealed that both PLWH with a positive serology for VZV and those who reported clinical symptoms of VZV reactivation had a more than threefold higher risk of experiencing any ischemic event.

Upon examining the individual characteristics of the six patients who reported ischemic events following HZ, we observed that all of them were male. Nearly all of these patients had previously been diagnosed with AIDS, and at the time of the ischemic event, none of them were on ART, except for one patient who was receiving a PI-based regimen. These findings align with what has already been documented in the existing literature [19].

A novelty in our study was to include peripheral thrombosis as a reported ischemic event. This is because we observed that in nearly half of the cases, patients with a history of ischemic events had also reported peripheral thrombosis, and these reports were almost evenly distributed between venous thrombosis in the lower limbs and arterial thrombosis affecting intestinal and pulmonary vessels.

Thrombotic complications following HZ have been described, especially in children, while a few case reports have described cerebral venous sinus thrombosis, deepvenous thrombosis of the lower limbs, and pulmonary embolism in adults [20,21,22,23,24,25].

While we could not establish a statistically significant difference, mainly due to the limited size of our study population, the distribution of types of ischemic events in our group of six patients with a history of HZ corresponded to what has been observed in other studies. Cerebrovascular ischemia was the most frequent presentation, followed by two cases of acute AMI and one case of peripheral thrombosis.

Further research is needed to gain a more profound understanding of the connection between VZV-associated vasculopathy and the disruption of the homeostasis/thrombosis balance, which can lead to peripheral thrombosis in both small and large blood vessels. In the case of HIV-infected patients, this challenge becomes even more complex, as the factors contributing to vascular diseases are numerous. They are related to the chronic inflammatory state associated with the underlying HIV disease, which tends to worsen with its duration and the type and duration of antiretroviral therapy (especially medications like protease inhibitors and abacavir), as well as factors such as the patient’s age, non-infectious comorbidities, and lifestyle [15]. It is worth emphasizing that all these variables were taken into account in our analysis. When we assessed the collective influence of VZV infection and its reactivation on elevating vascular risk in people living with HIV (PLWH), we observed that these factors became less prominent. The sole factor that significantly increased the risk of ischemic events, by nearly 10 times, was having a history of chronic heart disease.

Finally, there are limited available data regarding the role of vaccinations in preventing complications of HZ, particularly vascular and ischemic risks. As the prevalence of stroke is significantly higher in people who have not received vaccination compared to vaccinated individuals, some studies suggest that the protective effect of vaccinations may result from the reduced incidence of HZ in vaccinated individuals [26,27,28]. Unfortunately, our analysis was unable to provide clarification on this scientific question. This limitation should be noted, as we did not have information on vaccination status for the majority of our cohort due to the retrospective nature of our study.

Other study limitations that deserve to be mentioned are first, the small size of the study population, which, on the one hand, emphasizes the significance of our results, but on the other hand, limited our ability to assess important correlations, such as the relationship between specific types of ischemic events and VZV. Additionally, another limitation arising from the retrospective and observational nature of this study is the variation in the duration of follow-up among the two groups of patients. These durations were not predetermined but rather reflected the natural clinical history of the patients. A prospective study design would be more effective for assessing both the frequency and timing of ischemic events in patients with and without a history of VZV reactivation. Additionally, due to the lack of precise time data for some ischemic events, we were unable to determine whether the associated vascular risk related to VZV reactivation was short-term or long-term. Furthermore, because our study was conducted at a single center, the results should be interpreted with caution and applied primarily to the specific local context in which the study was conducted.

Notwithstanding the abovementioned limits, our research lays the groundwork for more comprehensive investigations into the potential role of herpes zoster as a vascular risk marker. To address this issue, we are committed to expanding our research with a larger sample, aiming to provide more conclusive insights into the VZV-vascular risk relationship. This endeavor will contribute to determining how a history of HZ can be integrated into the cardiovascular risk assessment of PLWH and to assess the long-term effectiveness of vaccines as a preventive measure in this context.

## 5. Conclusions

Similar to the general population, VZV infection, and in particular its reactivation, represents a factor that can increase vascular risk in individuals with HIV infection, especially in the presence of other risk factors, such as an underlying compromised heart condition. Prospective studies involving a larger sample of patients are needed to confirm our observations and assess whether interventions, including modifications to ART, co-medications and lifestyle changes, and potentially VZV vaccination, can mitigate the risk of ischemic events in this specific population.

## Figures and Tables

**Table 1:** Characteristics of the study population stratified by the history of physical manifestations of varicella–zoster virus (VZV) reactivation.

Variables	Overall (N = 581)	PLWH without VZV Reactivation (N = 546)	PLWH with VZV Reactivation (N = 45)	*p*-Value
Male sex, n (%)	471 (81)	438 (82)	33 (73)	0.16
Age, years, median (IQR)	48 (38–56)	47 (38–55)	56 (48–61)	*<0.001*
Non-Italian nationality, n (%)	63 (11)	57 811)	6 (13)	0.576
Transmission route, n (%)				*0.003*
Heterosexual contacts	214 (37)	200 (37)	14 (31)	
MSM	196 (34)	179 (33)	17 (38)	
IDU	40 (7)	31 (86)	9 (20)	
Other	126 (22)	121 (23)	5 (11)	
AIDS diagnosis, n (%)	230 (40)	203 (28)	27 (60)	*0.003*
Nadir CD4, cells/mL, median (IQR)	244 (109–286)	259 (121–401)	161 (87–262)	*<0.001*
Years of HIV diagnosis, median (IQR)	9 (6–14)	9 (5–13)	15 (9–27)	*<0.001*
Years of ART, median (IQR)	8 (5–11)	8 (5–11)	11 (8–20)	*<0.001*
History of abacavir, n (%)	152 (26)	135 (25)	17 (38)	0.06
Current ARV regimen, n (%)				*0.007*
2 NRTIs + NRTI	100 (17)	93 (17)	7 (16)	
2 NRTIs + PI	70 (12)	59 (11)	11 (24)	
2 NRTIs + INSTI	242 (42)	231 (43)	11 (24)	
NNRTI + INSTI	58 (10)	55 (10)	3 (7)	
PI + INSTI	8 (1)	4 (1)	2 (4)	
1 NRTI + INSTI	100 (17)	90 (17)	10 (22)	
Other	5 (1)	4 (1)	1 (2)	
Positive serology for VZV, n (%)	372 (74)	327 (61)	45 (100)	*<0.001*
Presence of co-morbidities, n (%)	294 (49)	250 (46)	34 (75)	<0.001
Hypertension	74 (26)	62 (25)	12 (35)	0.19
Dyslipidemia	160 (56)	140 (56)	20 (59)	0.75
Diabetes	43 (15)	38 (15)	5 (15)	0.94
Chronic heart disease	15 (5)	14 (6)	1 (3)	0.51
Other	93 (33)	81 (32)	12 (35)	0.74
Ischemic event, n (%)	31 (5)	25 (5)	6 (13)	*0.01*
AMI	9 (29)	7 (28)	2 (33)	0.21
Stroke	8 (25)	5 (20)	3 (50)	
Peripheral thrombosis	14 (45)	13 (52)	1 (17)	

IQR: Interquartile Range; IDU: Intravenous Drug User; MSM: Males Who Have Sex with Males; AIDS: Acquired Immunodeficiency Syndrome; ART: Antiretroviral Treatment; ARV: Antiretroviral; NRTIs: Nucleos(t)ide Reverse Transcriptase Inhibitors; NNRTIs: Non-Nucleos(t)ide Reverse Transcriptase Inhibitors; PIs: Protease Inhibitors; INSTIs: Integrase Strand Transfer Inhibitors; VZV: Varicella–Zoster Virus; AMI: Acute Myocardial Infarction; DVT: Deep Venous Thrombosis.

**Table 2:** Characteristics of six patients with a history of varicella–zoster virus (VZV) reactivation who reported any ischemic event (acute myocardial infarction, stroke, peripheral thrombosis, or a combination of these).

Patient	Sex	HBV/HCV Coinfection	Smoker	AIDS Diagnosis	Age at First VZV Reactivation (Years)	Clinical Manifestation of VZV Reactivation	On ART at Ischemic Stroke	Type of Ischemic Event
1	Male	No	No	Yes	67	Recurrent, multiple sites	No	Ischemic Heart Attack and Ischemic Brain Stroke
2	Male	No	No	Yes	-	Recurrent, multiple sites (vaccinated with Shinrix)	No	Ischemic Heart Attack and Ischemic Brain Stroke
3	Male	No	Yes	Yes	56	Single manifestation, Ramsay Hunt Syndrome	Yes (TAF/FTC/ DRV/cobi)	Ischemic Heart Attack
4	Male	No	-	Yes	34	Single manifestation, right arm	No	Thrombosis of Pulmonary Artery
5	Male	No	Yes	Yes	50	Recurrent, multiple sites	No	Ischemic Brain Stroke
6	Male	Yes (both)	Yes	No	55	Recurrent, multiple sites	No	Ischemic Brain Stroke

HBV: Hepatitis B Virus; HCV: Hepatitis C Virus; AIDS: Acquired Immunodeficiency Syndrome; VZV: Varicella–Zoster Virus; ART: Antiretroviral Treatment.

**Table 3:** Uni- and multivariate logistic regression estimating factors associated with a higher relative risk (odds ratio, O.R., with 95% confidence interval, C.I.) of acute myocardial infarction, stroke or deep venous thrombosis.

Variables	O.R. (95% C.I., *p*-Value)	aO.R. * (95% C.I., *p*-Value)
Male sex	0.78 (0.34–2.01, *p* = 0.58)	-
Age > 65 years	7.35 (2.98–17.01, *p* < 0.001)	2.76 (0.83–8.36, *p* = 0.08)
IDU	0.93 (0.15–3.25, *p* = 0.92)	-
Positive serology for VZV	4.01 (1.54–13.71, *p* = 0.01)	1.88 (0.56–7.54, *p* = 0.33)
History of HZ	3.14 (1.12–7.68, *p* = 0.018)	1.14 (0.14–6.16, *p* = 0.89)
Baseline CD4+ < 200 cells/mm^3^	1.37 (0.64–2.84, *p* = 0.40)	-
>10 years of HIV infection	1.37 (0.58–3.39, *p* = 0.47)	-
>10 years of ART	1.73 (0.35–31.33, *p* = 0.60)	-
History of abacavir	1.37 (0.60–2.91, *p* = 0.43)	-
Current PI-based regimen	0.70 (0.16–2.04, *p* = 0.56)	-
Hypertension	3.23 (1.41–7.40, *p* = 0.005)	2.84 (0.95–8.48, *p* = 0.06)
Type II diabetes	2.27 (0.84–5.59, *p* = 0.09)	1.88 (0.50–6.10, *p* = 0.32)
Dyslipidemia	1.06 (0.47–2.46, *p* = 0.88)	-
Chronic heart disease	11.51 (3.69–35.61, *p* < 0.001)	9.95 (2.15–45.46, *p = 0.003*)

O.R.: Odds Ratio; C.I.: Confidence Interval; aO.R.: Adjusted Odds Ratio; IDU: Intravenous Drug User; VZV: Varicella–Zoster Virus; ART: Antiretroviral Treatment. * Multivariate model includes all variables selected by backward selection that were retained with a *p*-value less than 0.2.

## Data Availability

The data presented in this study are available on request from the corresponding author. The data are not publicly available to ensure the protection of privacy.
